# Odor identification predicts the transition of patients with isolated RBD: A retrospective study

**DOI:** 10.1002/acn3.51615

**Published:** 2022-06-29

**Authors:** Tomoyuki Miyamoto, Masayuki Miyamoto

**Affiliations:** ^1^ Department of Neurology Dokkyo Medical University Saitama Medical Center Japan; ^2^ Department of Neurology Center of Sleep Medicine, Dokkyo Medical University Japan; ^3^ Dokkyo Medical University School of Nursing Japan

**Keywords:** Dementia with Lewy bodies, Lewy body disease, Parkinson's disease, REM sleep, behavior disorder, UPSIT‐40.

## Abstract

**Introduction:**

To determine if the severity of olfactory dysfunction in isolated REM sleep behavior disorder (IRBD) predicts conversion to Parkinson's disease (PD) or dementia with Lewy bodies (DLB).

**Methods:**

Olfaction was tested using the Japanese version of the University of Pennsylvania Smell Identification Test (UPSIT‐J) in 155 consecutive patients with polysomnography‐confirmed IRBD and 34 healthy controls. IRBD patients were followed up for 5.8 ± 3.2 (range 0.2–11) years. Thirty‐eight patients underwent repeat UPSIT‐J evaluation at 2.7 ± 1.3 years after the baseline test.

**Results:**

UPSIT‐J score was lower in IRBD patients than in age‐ and sex‐matched controls. The receiver operating characteristic curve analysis showed that the optimal cutoff score of 22.5 in UPSIT‐J discriminated between IRBD patients and controls with a sensitivity of 94.3% and specificity of 81.8%. Anosmia (UPSIT‐J score < 19) was present in 54.2% of IRBD patients. In total, 42 patients developed a neurodegenerative disease, of whom 17 had PD, 22 DLB, and 3 MSA. Kaplan–Meier analysis showed that the short‐term risk of Lewy body disease (LBD) was higher in patients with anosmia than in those without anosmia. At baseline, the UPSIT‐J score was similar between patients who developed PD and DLB (p = 0.136). All three IRBD patients (100%) who developed MSA did not have anosmia.

**Conclusions:**

In IRBD patients, anosmia predicts a higher short‐term risk of transition to LBD but cannot distinguish between PD and DLB. At baseline, preserved odor identification may occur in latent MSA. Future IRBD neuroprotective trials should evaluate anosmia as a marker of prodromal LBD.

## Introduction

The long‐term follow‐up of patients initially classified as idiopathic or isolated rapid eye movement sleep behavior disorder (IRBD) has shown that some patients develop Parkinson's disease (PD), dementia with Lewy bodies (DLB), or multiple system atrophy (MSA).^1^ A meta‐analysis of studies conducted in 13 countries on the risk of neurodegenerative diseases in IRBD patients showed a correlation between follow‐up duration and rate of progression.^2^ The risk of development of clinically defined Lewy body disease (LBD) is almost 20.3% at 5 years and 51.4% at 10 years after the IRBD diagnosis.^3^ Thus, IRBD patients may be enrolled in neuroprotective trials to determine the effects on the neurodegenerative process.^4^, ^5^ For such neuroprotective trials, markers of short‐term risk of clinically defined LBD in IRBD patients are needed to monitor the neurodegenerative process.^1^ Abnormal α‐synuclein deposition occurs early in the neurodegenerative process across the central and peripheral nervous systems and might precede the appearance of motor symptoms and cognitive decline by several decades. In clinical practice, abnormal α‐synuclein deposition has been detected in glands, skin, or CSF, which is attracting attention as a biomarker.^6^ A previous meta‐analysis reported that many IRBD patients have prodromal clinical markers of PD or DLB, such as hyposmia.^7^, ^8^ Previous studies suggested that hyposmia occurs in IRBD patients^9^, ^10^, ^11^ and may identify patients with a risk of early conversion to a clinically defined synucleinopathy, especially LBD.^12^, ^13^

In the present study of a large IRBD Japanese cohort with long‐term follow‐up, we retrospectively investigated the severity of odor identification dysfunction as a marker of short‐term phenoconversion to LBD, such as PD or DLB.

## Methods

### Participants

Odor identification was evaluated in 155 IRBD patients diagnosed at the Sleep Center Tochigi, Japan, between October 2009 and April 2017 using the Japanese version of the 40‐item University of Pennsylvania Smell Identification Test™ (UPSIT‐J)^14^, ^15^ and Odor Stick Identification Test for Japanese (OSIT‐J, Daiichi Yakuhin, Co. Ltd., Tokyo, Japan),^16^ (Supplementary Fig. 1).

IRBD was diagnosed based on the history of dream‐enacting behavior, video‐polysomnographic demonstration of increased electromyographic activity linked to abnormal behaviors in REM sleep, and absence of motor and cognitive impairment, according to the International Classification of Sleep Disorders, Second Edition.^17^ Surface EMG electrodes were placed on the chin and limbs (bilateral tibialis anterior muscles) to evaluate the increased EMG activity during REM sleep. The chin and limb EMG channels were used to evaluate the scoring and evaluation of the increased EMG activity during REM sleep. The limb movement and abnormal behaviors during REM sleep were confirmed using the simultaneously recorded video‐PSG.

#### Olfactory assessment

UPSIT‐J is a scratch‐and‐sniff test where each item has one correct and three incorrect answers. This test takes 15 min to administer and involves the identification of 40 microencapsulated odors in a forced‐choice format (Supplementary Fig. 2). The identification score is equal to the number of correct answers (range: 0–40 points), with higher scores corresponding to better odor identification. We classified the olfactory dysfunction using the data for healthy individuals published by Doty et al.^15^ as follows: 0–5, probable malingering; 6–18, total anosmia; 19–25, severe microsmia; 26–29, moderate microsmia (males); 26–30, moderate microsmia (females); 30–33, mild microsmia (males); 31–34, mild microsmia (females); 34–40, normosmia (male); and 35–40, normosmia (female). In this study, anosmia was defined as a score < 19^15^ (Table 1).

**Table 1:** Olfactory diagnosis for UPSIT‐J score in IRBD (n = 155).

Olfactory diagnosis	UPSIT score*	number	%
Anosmia	6–18	84	54.2
Severe microsmia	19–25	51	32.9
Moderate microsmia M	26–29	12	7.7
Moderate microsmia F	26–30	2	1.3
Mild microsmia M	30–33	4	2.6
Mild microsmia F	31–34	1	0.6
Normosmia M	34–40	1	0.6
Normosmia F	35–40	0	0.0

F, female; IRBD, isolated REM sleep behavior disorder; M, male; UPSIT, unified Pennylvania smell identification test. *, Doty RL, The Smell Identification Test, TM administration manual, P 7, 3rd edn. Sensonics Inc., Philadephia, 1995.^15^

On the other hand, UPSIT‐J and normative data are not available for the Japanese population. Importantly, 10 items are different between the original version of UPSIT^14^, ^15^ and UPSIT‐J^16^ as follows; lemon, cherry, clove, pine, liquorice, fruit punch, paint thinner, root beer, lime, and grape is an original version‐only item, and grapefruit, incense stick (sandalwood), citron (yuzu fruit), baby powder, apple, rubber tire, popcorn, garlic, fish, and coffee is a Japanese version only item (Table 2). To improve the accuracy, we assessed the receiver operating characteristic (ROC) curves and area under curve (AUC) in differentiating IRBD from healthy individuals (Mini‐Mental State Examination (MMSE)^18^ > 24, REM sleep behavior screening questionnaire for Japanese^19^ < 5) in this study. In total, 34 age‐ and gender‐matched healthy individuals and 34 IRBD patients were enrolled in the UPSIT‐J cut‐off study (Table 3). These 34 IRBD patients were selected from 155 IRBD patients. ROC analysis was used to determine the UPSIT‐J cutoff values to distinguish between the two groups (healthy controls versus IRBD patients) (Supplementary Fig. 3).

**Table 2:** Correct odor identification rates in the Japanese version of the University of Pennsylvania Smell Identification Test for age‐ and sex‐matched healthy controls (n = 34) and IRBD patients (n = 34).

No.	Item	Control	IRBD	p‐value	No.	Item	Control	IRBD	p‐value
1	Pizza	58.8	29.4	0.015	21	Lilac	88.2	35.3	< 0.001
2	Bubble gum	91.2	29.4	< 0.001	22	Turpentine	76.5	47.1	0.013
3	Menthol	100.0	64.2	< 0.001	23	Peach	88.2	41.2	< 0.001
4	*Grapefruit*	97.1	50.0	< 0.001	24	*Rubber tire*	52.9	17.6	0.002
5	**Motor oil**	67.6	47.1	**0.086**	25	**Dill pickle**	41.2	23.5	**0.120**
6	Mint	85.3	61.8	0.028	26	Pineapple	85.3	50.0	0.002
7	Banana	82.4	38.2	< 0.001	27	*Popcorn*	70.6	44.1	0.027
8	***Incense stick (Sandalwood)***	76.5	76.5	**1.000**	28	Orange	76.5	50.0	0.024
9	Cowhide (Leather)	91.2	58.8	0.002	29	Poultice	100	67.6	< 0.001
10	Coconut	80.0	47.1	0.002	30	Watermelon	85.3	50.0	0.002
11	Onion	94.1	70.6	0.011	31	*Garlic*	97.1	76.5	0.012
12	***Yuzu fruit***	14.7	5.9	**0.231**	32	Grass	50.0	17.6	0.005
13	*Baby powder*	94.1	58.8	< 0.001	33	Smoke	91.2	55.9	< 0.001
14	Cheese	61.8	29.4	0.007	34	***Fish***	55.9	32.4	**0.051**
15	Cinnamon	70.6	47.1	0.049	35	Grape	58.8	20.6	0.001
16	Gasoline	70.6	44.1	0.027	36	***Coffee***	85.3	64.7	**0.050**
17	Strawberry	52.9	14.7	< 0.001	37	Soap	82.4	47.1	0.002
18	Cedar	76.5	41.2	0.003	38	Natural gas	91.2	35.3	< 0.001
19	**Chocolate**	76.5	61.8	**0.189**	39	**Rose**	52.9	32.4	**0.086**
20	*Apple*	82.4	32.4	< 0.001	40	Peanut	91.2	44.1	< 0.001

Value, %; IRBD, isolated REM sleep behavior disorder; p‐value, Pearson's Chi‐squared test p‐value for the difference between the percentages of controls and IRBD patients who identified item. Bold items had no significant difference.

Lemon, cherry, clove, pine, liquorice, fruit punch, paint thinner, root beer, lime, and grape were only included in the original version, whereas grapefruit, incense stick (sandalwood), yuzu fruit, baby powder, apple, rubber tire, popcorn, garlic, fish, and coffee were only included in the Japanese version (italicized).

**Table 3:** Background characteristics of the healthy control and age‐ and sex‐matched selected IRBD patients.

	Heathy controls	IRBD patients	p‐value
N	34	34	N/A
Age, years	64.0 ± 5.3	64.3 ± 5.3	0.65
Sex (males/females)	20/14	20/14	N/A
MMSE	28.6 ± 1.6	27.9 ± 2.0	0.06
RBDSQ‐J	1.5 ± 1.0	6.9 ± 3.3*	p < 0.001
UPSIT‐J	30.7 ± 4.3	17.6 ± 5.3	p < 0.001
OSIT‐J	9.9 ± 1.3	2.1 ± 1.4	p < 0.001

IRBD, isolated REM sleep behavior disorder; MMSE, Mini Mental State Examination; OSIT‐J, Odor Stick Identification Test for Japanese; RBDSQ‐J, REM sleep behavior screening scale for Japanese; UPSIT‐J, Japanese version of the 40‐item University of Pennsylvania Smell Identification Test™; p‐value, Mann–Whitney Test; N/A, not available; *, n = 22.

In previous studies, IRBD patients were evaluated using a shorter odor identification test.^9^, ^10^, ^11^ We also investigated whether short versions of the odor identification test could make short‐term predictions of phenotypic conversion. A short version of the test used in the present study was evaluated using the previously reported OSIT‐J.^11^ OSIT‐J is composed of 12 odorants familiar to the Japanese population, ^20^ namely condensed milk, cooking gas, curry, hinoki (Japanese cypress wood), Indian ink, Japanese orange, menthol, perfume, roasted garlic, rose, socks smelling of sweat, and wood. For each odorant, the participant was asked to select the correct from a card showing four names of odors. If the participant could not choose one of the four options, he/she must respond by selecting 1 of the 2 answers: “detectable but not recognizable” or “no odor detected”. By adding two optional answers, the ambiguous answers are excluded and subjects are forced to provide more accurate assessments than when four forced choices are provided. In the previous study by Ogihara et al,^21^ the OSIT‐J scores showed a significant correlation with those of the UPSIT‐J (r = 0.86, p < 0.0001, n = 104). In our previous study, the OSIT‐J scores had a moderate correlation with those of the UPSIT‐J (control subjects, r = 0.5032, P = 0.017, n = 22; IRBD, r = 0.6842, p < 0.0001, n = 29).^22^ The total number of correct answers for the 12 odorants presented is the OSIT‐J score. In this study, functional anosmia was defined as an OSIT‐J score ⩽ 4.^23^

#### Longitudinal assessment

The odor identification test at baseline was performed in 155 IRBD patients, who were followed up every 1–3 months at the Sleep Center. During these visits, IRBD patients were assessed for the development of neurodegenerative diseases, including PD 19,^24^ DLB,^25^ and MSA.^26^ MIBG data were used as an adjunct to the diagnosis of neurodegenerative diseases (PD or DLB vs. MSA). Lewy body disease (PD or DLB) was diagnosed using ^123^I‐metaiodobenzylguanidine myocardial scintigraphy (^123^I‐MIBG) and the lower limit of the heart‐to‐mediastinum ratio for early and delayed images was set to 2.2 according to the database of the Standardization Working Group of the Japanese Society of Nuclear Medicine.^27^

In October 2020 (end of the study), we assessed whether patients remained disease‐free or developed PD, DLB, or MSA (Table 4).

**Table 4:** Outcome of IRBD patients.

Outcome		UPSIT ≥23	UPSIT <23	p‐value
	155	34 (21.9)	121 (78.1)	**< 0.001**
Disease‐free	113	25 (22.1)	88 (77.9)	**< 0.001**
Converted to PD	17	3 (17.6)	14 (82.4)	**0.013**
Converted to DLB	22	3 (13.6)	19 (86.4)	**< 0.001**
Converted to MSA	3	3 (100)	0 (0)	0.250

		UPSIT ≥19	UPSIT <19	p‐value
	155	71 (45.8)	84 (54.2)	0.335
Disease‐free	113	57 (50.4)	56 (49.6)	1.00
Converted to PD	17	4 (23.5)	13 (76.5)	0.49
Converted to DLB	22	7 (31.8)	15 (68.1)	0.134
Converted to MSA	3	3 (0)	0 (0)	0.250

DLB, dementia with Lewy bodies; IRBD, isolated rapid eye sleep behavior disorder; PD, Parkinson's disease; MSA, multiple system atrophy. p‐value, binomial test (two‐tail p‐value). Bold results were statistically significant.

After baseline olfactory assessment, 38 IRBD patients selected randomly underwent repeat UPSIT‐J evaluations for the interval mean of 2.7 ± 1.3 years (Table 5).

**Table 5:** Repeat UPSIT assessment (n = 38).

	Age	Sex	UPSIT score (1st)	Anosmia (n = 18); non‐anosmia (n = 20)	UPSIT score (2nd)	Anosmia (n = 24); non‐anosmia (n = 14)	Absolute differences (2nd − 1st)	Interval between 1st and 2nd, years	Interval between 1st and outcome, years	Interval between 2nd and outcome, years	Phenoconversion type
Patient 1	63	M	28	non‐anosmia	15	anosmia	−13	5.2	5.8	0.6	DLB
Patient 2	77	F	18	anosmia	7	anosmia	−11	2.0	4.0	2.0	disease free
Patient 3	62	M	24	non‐anosmia	14	anosmia	−10	5.1	11.0	5.9	disease free
Patient 4	57	M	17	anosmia	10	anosmia	−7	2.2	3.2	1.0	DLB
Patient 5	62	M	21	non‐anosmia	14	anosmia	−7	2.2	9.0	6.8	disease free
Patient 6	72	M	13	anosmia	7	anosmia	−6	4.3	10.9	6.6	disease free
Patient 7	64	F	21	non‐anosmia	15	anosmia	−6	2.5	9.0	6.5	disease free
Patient 8	71	M	20	non‐anosmia	14	anosmia	−6	3.1	8.5	5.4	disease free
Patient 9	71	F	22	non‐anosmia	16	anosmia	−6	1.0	1.2	0.2	disease free
Patient 10	61	M	19	non‐anosmia	14	anosmia	−5	2.7	10.1	7.4	disease free
Patient 11	77	M	15	anosmia	11	anosmia	−4	4.3	−4.0	−8.3	PD*
Patient 12	69	M	16	anosmia	13	anosmia	−3	2.1	8.8	6.7	disease free
Patient 13	77	M	16	anosmia	13	anosmia	−3	2.0	8.5	6.5	disease free
Patient 14	69	M	21	non‐anosmia	18	anosmia	−3	2.0	8.1	6.1	disease free
Patient 15	63	M	22	non‐anosmia	20	non‐anosmia	−2	4.9	6.4	1.6	DLB
Patient 16	65	M	20	non‐anosmia	18	anosmia	−2	2.2	5.6	3.4	DLB
Patient 17	65	M	29	non‐anosmia	27	non‐anosmia	−2	1.2	−0.3	−1.6	PD**
Patient 18	61	M	22	non‐anosmia	20	non‐anosmia	−2	4.1	9.7	5.6	disease free
Patient 19	62	M	30	non‐anosmia	28	non‐anosmia	−2	2.2	8.3	6.1	disease free
Patient 20	71	M	18	anosmia	17	anosmia	−1	4.0	7.6	3.5	DLB
Patient 21	70	M	30	non‐anosmia	29	non‐anosmia	−1	2.1	8.6	6.5	MSA
Patient 22	74	M	19	non‐anosmia	18	anosmia	−1	4.8	10.7	5.9	disease free
Patient 23	71	M	15	anosmia	14	anosmia	−1	2.0	7.9	5.9	disease free
Patient 24	71	M	15	anosmia	14	anosmia	−1	1.1	7.2	6.1	disease free
Patient 25	71	M	23	non‐anosmia	23	non‐anosmia	0	2.0	4.8	2.8	MSA
Patient 26	65	F	14	anosmia	14	anosmia	0	1.2	3.3	2.1	disease free
Patient 27	68	F	16	anosmia	17	anosmia	1	2.1	5.5	3.4	PD
Patient 28	71	M	27	non‐anosmia	28	non‐anosmia	1	2.2	6.7	4.5	PD
Patient 29	51	M	26	non‐anosmia	27	non‐anosmia	1	1.1	7.2	6.1	disease free
Patient 30	64	M	34	non‐anosmia	35	non‐anosmia	1	4.3	10.3	6.0	disease free
Patient 31	64	M	18	anosmia	19	non‐anosmia	1	4.1	8.2	4.1	disease free
Patient 32	75	F	14	anosmia	16	anosmia	2	2.0	3.0	1.0	DLB
Patient 33	68	M	18	anosmia	20	non‐anosmia	2	4.6	9.4	4.8	PD
Patient 34	61	M	16	anosmia	19	non‐anosmia	3	2.1	8.4	6.3	disease free
Patient 35	65	M	21	non‐anosmia	25	non‐anosmia	4	1.2	7.2	6.0	disease free
Patient 36	78	M	13	anosmia	18	anosmia	5	2.2	7.7	5.5	disease free
Patient 37	73	M	13	anosmia	19	anosmia	6	2.0	7.6	5.7	PD
Patient 38	56	F	16	anosmia	25	non‐anosmia	9	4.2	6.3	2.1	PD
Mean	67.2	M:31	20.0		18.2		−1.8	2.7	6.9	4.1	
SD	6.3	F:7	5.4		6.3		4.6	1.3	3.2	3.1	

Anosmia: < 19; non‐anosmia: 19–40; dementia with Lewy bodies, DLB; multiple system atrophy, MSA; Parkinson's disease, PD; standard deviation, SD; the Japanese version of unified Pennsylvania smell identification test, UPSIT‐J. *, UPSIT assessment was performed after developed PD.

This study was approved by our institutional ethics committee at Dokkyo Medical University (approval number R‐2‐22) and performed in accordance with the ethical standards laid down in the 1964 Declaration of Helsinki and its later amendments.

### Statistical analysis

Comparisons between groups were performed using χ^2^ test and Mann–Whitney U test as appropriate. Neurological disease‐free survival rates were estimated using Kaplan–Meier analysis and Cox‐proportional hazards analysis. Disease‐free survival rates were assessed from the date of UPSIT‐J or OSIT‐J to the date of PD, DLB, or MSA diagnosis, or the last follow‐up for censored observations.

Repeated measures χ^2^ test was used to evaluate the differences in UPSIT‐J score between baseline and repeat evaluations at 2.7 ± 1.3 years. P‐values <0.05 were considered statistically significant.

## Results

### Cut‐off study

The study included 34 healthy controls matched for age with IRBD patients (Table 3). The healthy controls had a MMSE score of 28.6 ± 1.7 and included 4 (11.8%) current smokers and 15 (44.1%) ex‐smokers. Among the healthy controls, the RBDSQ‐J^14^ score was 1.5 ± 1.0 (0–4). The OSIT‐J score was lower in patients than in controls (2.1 ± 1.4 vs. 9.9 ± 1.3, respectively; p < 0.000). UPSIT‐J score was lower in IRBD patients than in healthy controls (17.6 ± 5.4 vs. 30.7 ± 4.3, respectively; p < 0.001). The optimal cutoff score for UPSIT‐J to discriminate IRBD was <22.5 and had a sensitivity of 94.3% and specificity of 81.8% (Supplementary Fig. 3). With regard to the 32 out of 40 odorants, the correct answers were significantly different between controls and IRBD patients (P < 0.05, Table 2). The answers for bubble gum, gasoline, lilac, apple, grapefruit, peach, peanut, and banana were significant discriminators between controls and IRBD patients (AUC >0.70, p = 0.00). The correct answer rate for yuzu fruit, dill pickles, grass, strawberry, rubber tire, rose, and fish were low (less than 60%) in healthy controls. The answers for coffee, rose, chocolate, fish, dill pickle, incense stick, and yuzu fruit were not significant discriminators between controls and IRBD patients.

### Follow‐up assessment

We followed up 155 IRBD patients (81.9% males; age: 67.4 ± 6.5 years, range: 46–87) for 5.6 ± 3.2 (range: 0.2–11) years. Anosmia (UPSIT‐J score < 19) and hyposmia (UPSIT‐J score < 23) were present in 84 (54.2%) and 121 (78.1%) patients at baseline, respectively. At the end of the study, 42 patients (27.1%) developed clinically defined synucleinopathy, including PD (n = 17), DLB (n = 22), and MSA (n = 3). The baseline UPSIT‐J score was similar between patients who developed PD and DLB (16.7 ± 5.8 vs. 16.6 ± 6.5, respectively; p = 0.916). Hyposmia was present in 14 out of 17 patients (82.4%) who developed PD and 19 out of 22 (86.4%) who developed DLB, respectively. The three IRBD patients who developed MSA had normal UPSIT‐J score at baseline (27.3 ± 3.8). (Table 4).

**Figure 1:**
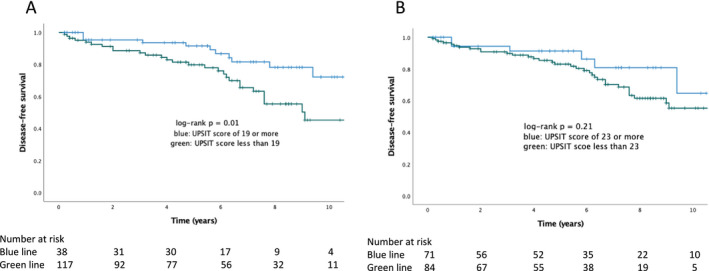
Progression of the onset of Lewy body disease in patients with isolated rapid eye movement sleep behavior disorder (Kaplan–Meier analysis). [Colour figure can be viewed at wileyonlinelibrary.com]

Patients with anosmia (UPSIT‐J score < 19) converted to LBD (PD or DLB) in a significantly shorter time duration than those without anosmia (log‐rank test, p < 0.01; Fig. 1A). In the anosmia group, the estimated 3‐, 5‐, 7‐, and 9‐year risks of developing LBD (PD or DLB) were 12.7%, 20.2%, 34.4%, and 49.7%, respectively. After adjusting for age and sex, Cox proportional‐hazards analysis showed that anosmia on UPSIT‐J (hazard ratio = 2.167; 95% confidence interval = 1.071–4.386; p < 0.032) predicted progression to LBD (PD or DLB). Conversely, patients with a UPSIT‐J score of less than 23 did not have a significantly shorter time duration of conversion to LBD (PD or DLB) compared to patients with a UPSIT‐J score of 23 or more (log‐rank test, p = 0.201, Fig. 1B).

Thirty‐eight patients, selected randomly, with a mean age of 67.3 ± 6.3 years underwent two evaluations with a mean interval between evaluations of 2.7 ± 1.3 years. In the two patients who developed MSA, anosmia was not detected on the first and second visits, and the odor identification did not decrease over time. Of the six patients who developed DLB, three had anosmia whereas the remaining three did not have anosmia. Two of the three patients without anosmia developed anosmia on the second visit and showed a decline in odor identification over time. Of the seven patients who developed PD, five had anosmia and two did not have anosmia at baseline. Two of the five cases with anosmia at baseline, who had PD but not dementia, did not have anosmia at the second visit. At baseline, among the two patients who progressed to PD, one had anosmia, whereas the other did not have anosmia. The second evaluation was performed after PD onset, but a decrease in odor identification was observed over the years. There were 23 cases of disease‐free, 10 cases of anosmia in the first visit, and anosmia in the second visit. Of the 23 patients who remained disease‐free, 10 had anosmia at the first and second visits. Of the 13 patients that had no anosmia at the first visit. Eight of 13 cases became anosmia at the second visit and showed a decline in odor identification over time (Table 5).

Anosmia, defined as OSIT‐J score ⩽ 4, did not have a significantly shorter time duration of conversion to LBD (PD or DLB) compared to patients in the non‐anosmia group (OSIT‐J score more than 4) (log‐rank test, p = 0.401). When adjusted for age, sex, and OSIT‐J score ⩽ 4, Cox proportional‐hazards analysis showed that age (hazard ratio = 1.066; 95% confidence interval = 1.012–1.122; p < 0.016) predicted progression to LBD (PD or DLB), but OSIT‐J score ⩽ 4 did not (p = 0.378).

## Discussion

In this large cohort of IRBD patients with a long‐term follow‐up of almost 11 years, we found that the severity of odor identification dysfunction at baseline predicted an increased short‐term risk of phenoconversion to LBD, such as PD and DLB. However, the severity of olfactory dysfunction does not individually predict conversion to PD or DLB. The three IRBD patients who subsequently developed MSA had normal olfactory identification, whereas 84.6% of those who developed LBD had anosmia/hyposmia at baseline. All three patients with prodromal MSA had consistently normal findings. Therefore, the presence of anosmia/hyposmia in IRBD patients is more likely to be related to underlying LBD than MSA, which is in agreement with a previous report.^28^ Thus, the severity of olfactory dysfunction helps to identify IRBD patients at high risk of phenoconversion to PD or DLB. Conversely, OSIT‐J, which was performed by evaluating 12 types of Japanese population who have an affinity for life, did not predict early phenoconversion to PD or DLB.

UPSIT is a validated, easily administered test that is widely used in the evaluation of neurodegenerative disorders, such as PD or DLB.^29^, ^30^, ^31^ However, UPSIT has rarely been evaluated in a large number of patients with IRBD, which is prodromal to PD^10^. The mean UPSIT score in the IRBD cohort (17.7 points) was similar to that of a PD cohort evaluated in a recent meta‐analysis.^8^ When the studies were subclassified according to the years of PD duration, no significant differences were observed in the UPSIT score. This indicates that olfactory loss is present in IRBD patients at a similar severity to PD patients, and the olfactory loss remained stable over time, probably reflecting a floor effect. In addition, the severity of dysosmia was related to changes in brain morphology; orbitofrontal volume reduction in anosmic IRBD is related to the UPSIT score.^32^ Serial DAT‐SPECT imaging of the nigrostriatal dopaminergic system^33^ and assessment of motor and cognitive functions^1^ show deterioration over time in IRBD patients. However, cardiac MIBG scintigraphy^34^ and substantia nigra hyperechogenicity of the midbrain on transcranial sonography^35^ are stable markers. UPSIT is a widely used tool for the assessment of olfaction that has been reported to have a high test‐retest reliability at 2‐week intervals (r = 0.95) and 6‐month intervals (*r* = 0.92) in young and middle‐aged adults with intact cognition. Repeated evaluations of the UPSIT score may distinguish between diseases based on the severity of odors identification dysfunction, which is less affected by the learning effect.^36^ In a previous study by Iranzo et al., serial UPSIT‐J scores did not change over time.^37^ In another study,^38^ olfaction and color vision declined as long as 5 years before diagnosis, with only a slight decline in preclinical stages. In the present study, the UPSIT‐J score decreased over time, especially among IRBD patients that progressed to DLB compared to those that progressed to PD (Table 4), suggesting a relationship between UPSIT‐J score with cognitive decline. Therefore, repeated UPSIT‐J scores may predict disease subtypes, such as DLB or Parkinson's disease dementia (PDD). Baba et al.^23^ found that hyposmia, a typical non‐motor feature of PD, predicted PDD. In that study, the multivariate logistic analysis identified severe hyposmia and visuoperceptual impairment as independent risk factors of subsequent dementia within 3 years. In a previous study, the relative risk of LBD in the lowest tertile of olfactory function was 7.3 (95% confidence interval: 1.8–29.6) compared to the top two tertiles.^12^ In the discrete time‐survival analyses of the follow‐up of normal elderly individuals (n = 757), lower baseline UPSIT scores were strongly associated with the transition to AD dementia (hazard ratio [HR] 1.099 per point interval; 95% CI 1.067–1.131). This association of lower baseline UPSIT scores with AD dementia remained highly significant (HR 1.072 per point interval; 95% CI 1.036–1.109) after the inclusion of sex, age, test language, education, Selective Reminding Test‐TR, and functional impairment as covariates in the model. These findings suggest that the severity of the odor identification may predict cases of neurogenerative dementia.^39^

The UPSIT is a 40‐item test and is one of the most commonly used smell tests worldwide. Shorter odor identification tests have also been developed, either as standalone (version of 12‐item Brief Smell Identification test [BSIT]) or preliminary (versions of 4‐item Pocket Smell Test [PST]) tests. The 12‐item BSIT performed better than the full 40‐item test in a discovery cohort, but not in an independent replication cohort.^40^ These findings have several practical implications for the use of olfactory tests for PD: the 12‐item BSIT is sufficient for the evaluation and may be time‐ and cost‐saving compared to the full UPSIT. However, in the present study, progression was predicted by the severity of dysfunction on 40‐item UPSIT‐J, which evaluated more items than the 12‐item OSIT‐J. The brief version of the odor identification test, e.g. 12‐item OSIT‐J or 12‐item UPSIT subset, is useful as a diagnostic marker of IRBD, but items were removed from the 40‐item subsets suggesting that significantly shorter tests would lack sufficient diagnostic utility for predicting the short‐term risktransition from IRBD to PD or DLB.

The combination of hyposmia and abnormal dopamine transporter imaging in this cohort of 280 hyposmic and normosmic individuals was associated with a phenoconversion rate of 25%, compared to a rate of 2.5% using hyposmia alone, highlighting the potential of hyposmia as a combined biomarker.^41^ In our other recent study, the combination of olfactory dysfunction, substantia nigra hyperechogenicity,^42^ and cerebral perfusion studies are useful for predicting conversion to neurodegenerative dementia.^43^

In 34 out of 155 (21.9%) IRBD patients with UPSIT ≥23, which is the cut‐off value that discriminates between IRBD patients and healthy people at baseline, 25 out of 34 (73.5%) remained disease‐free during follow‐up. Among 9 out of 34 (26.5%) IRBD patients, 3 who developed MSA, 3 who developed PD, and 3 who developed DLB had preserved olfaction before conversion. Few IRBD patients with preserved sense of smell converted to PD or DLB (Table 4, Supplementary Fig. 4). Based on these results, it is necessary to distinguish MSA patients from the small number of PD or DLB patients with a preserved sense of smell. In these cases, in addition to the odor identification test, differential symptoms^44^ that can be used for the clinical diagnosis of MSA include early autonomic symptoms,^45^ stridor or sleep‐breathing disorder,^46^ cardiac MIBG scintigraphy test,^47^ hyperechogenicity of midbrain substantia nigra,^48^ and MRI findings.^49^

The study had a few limitations. Japanese individuals are unfamiliar with a few odors used in UPSIT‐J, which may have led to a cultural bias for some test odorants. In addition, pathological confirmation for neurodegenerative disorders was not obtained. However, our study also had some strengths, including the use of specific differential clinical diagnostic markers for PD, DLB, and MSA, such as MIBG scintigraphy for the assessment of phenoconversion. In addition, odor identification using UPSIT‐J was used in a large video‐polysomnographic confirmed IRBD cohort with long‐term follow‐up.

In conclusion, the severity of odor identification dysfunction, which is a lower baseline UPSIT‐J score, in IRBD patients is useful to identify individuals at high short‐term risk of phenoconversion to PD or DLB.

## Conflict of Interest

None of the authors report a potential conflict of interest related to this work.

## Supporting information


**Supplementary Figure 1** Study flow chart of the follow‐up of patients with an isolated rapid eye movement disorder (IRBD). DLB, dementia with Lewy bodies; MMSE, Mini‐Mental State Examination; Odor Stick Identification Test for Japanese, OSIT‐J; PD, Parkinson's disease; PSG, polysomnography; the Japanese version of the 40‐item University of Pennsylvania Smell Identification Test™, UPSIT‐J; the Japanese version of the REM sleep behavior disorder screening questionnaire, RBDSQ‐J; UPDRS, Unified Parkinson's Disease Rating Scale.

**Supplementary Figure 2** The Japanese version of the 40‐item University of Pennsylvania Smell Identification Test™.

**Supplementary Figure 3** ROC curves of the 40‐item University of Pennsylvania Smell Identification Test™ to distinguish isolated rapid eye movement disorder patients from healthy controls.

**Supplementary Figure 4** Outcome and duration of follow‐up of isolated rapid eye movement disorder.
