# Supplementary figures and images for: Odor identification predicts the transition of patients with isolated RBD: A retrospective study
Source: Ann Clin Transl Neurol. 2022 Jun 29;9(8):1177–85. doi: 10.1002/acn3.51615 (PMC9380141; doi:10.1002/acn3.51615)

Supplementary Figure 1

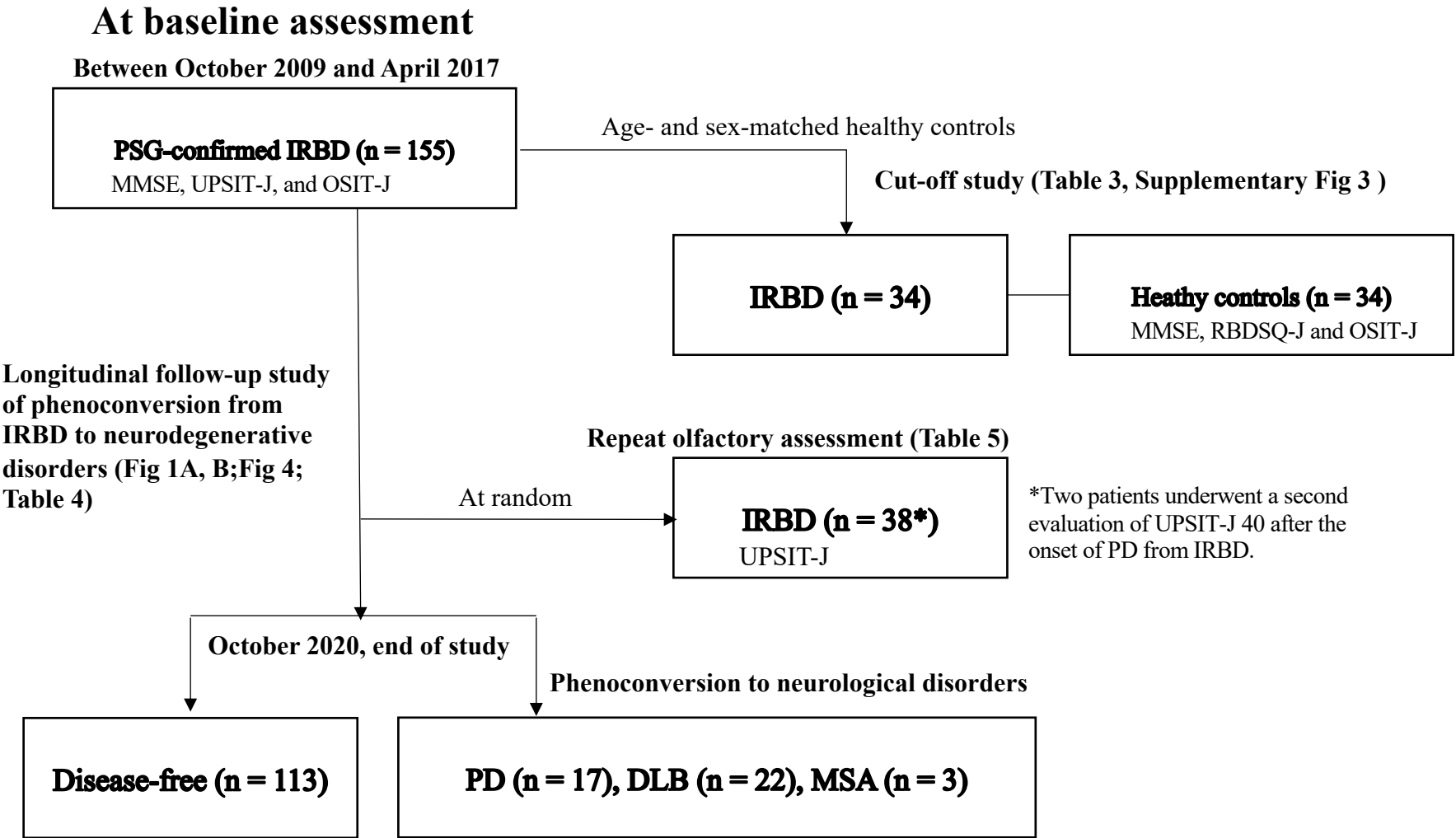

Supplement: Supplementary file 1 — Supplementary Figure 1 Study flow chart of the follow‐up of patients with an isolated rapid eye movement disorder (IRBD). DLB, dementia with Lewy bodies; MMSE, Mini‐Mental State Examination; Odor Stick Identification Test for Japanese, OSIT‐J; PD, Parkinson's disease; PSG, polysomnography; the Japanese version of the 40‐item University of Pennsylvania Smell Identification Test™, UPSIT‐J; the Japanese version of the REM sleep behavior disorder screening questionnaire, RBDSQ‐J; UPDRS, Unified Parkinson's Disease Rating Scale. [file ACN3-9-1177-s004.pdf]

Supplementary Figure 2

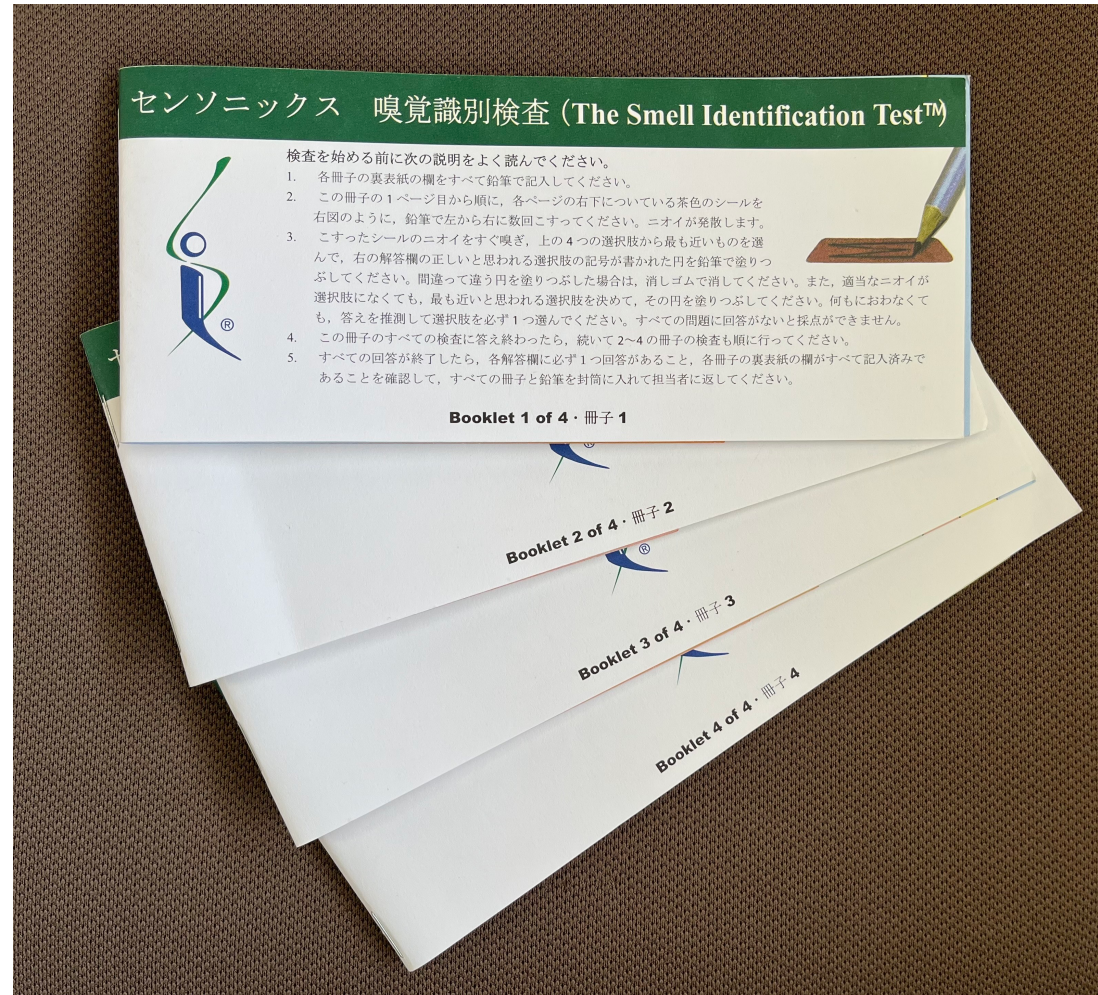

Supplement: Supplementary file 2 — Supplementary Figure 2 The Japanese version of the 40‐item University of Pennsylvania Smell Identification Test™. [file ACN3-9-1177-s001.pdf]

Supplementary Figure 3

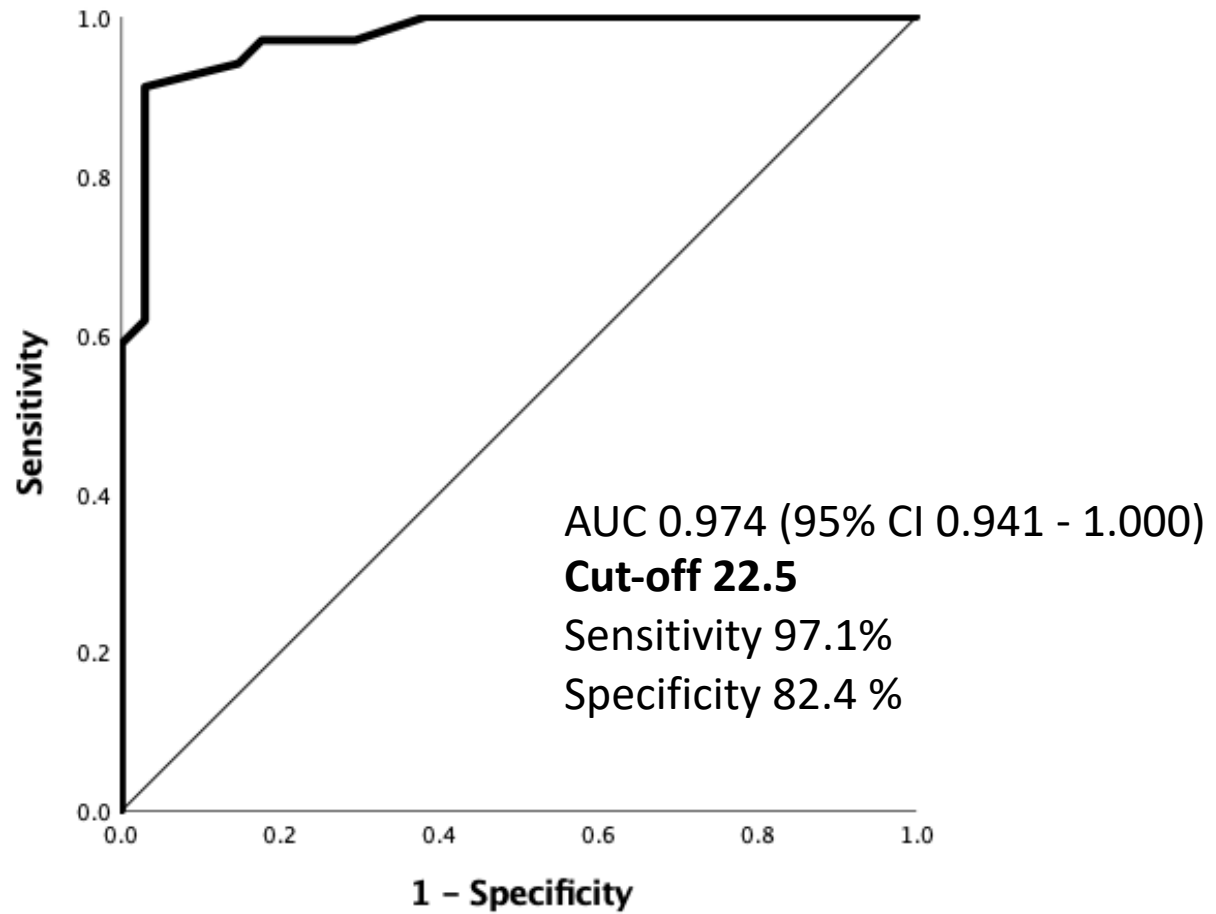

Supplement: Supplementary file 3 — Supplementary Figure 3 ROC curves of the 40‐item University of Pennsylvania Smell Identification Test™ to distinguish isolated rapid eye movement disorder patients from healthy controls. [file ACN3-9-1177-s002.pdf]

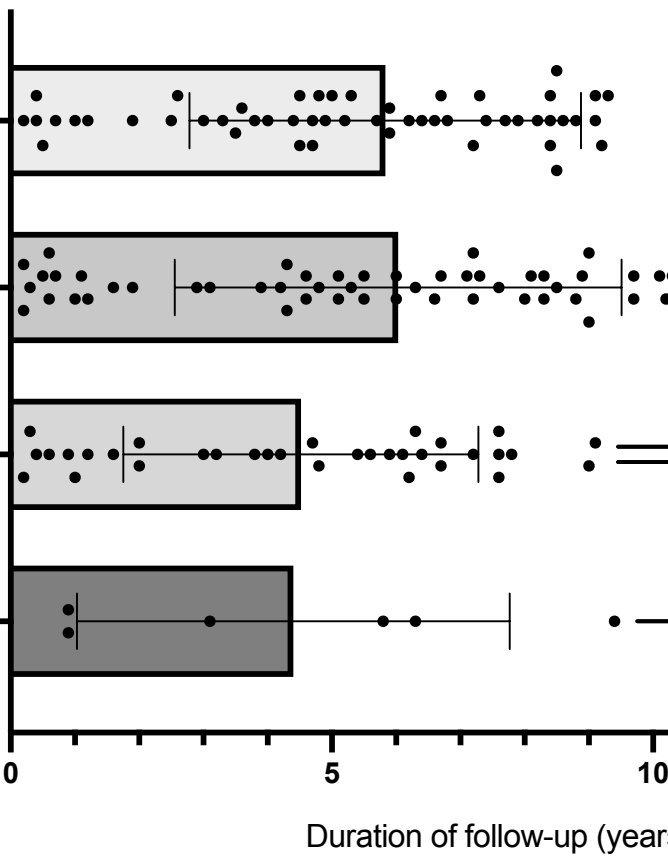

Supplement: Supplementary file 4 — Supplementary Figure 4 Outcome and duration of follow‐up of isolated rapid eye movement disorder. [file ACN3-9-1177-s003.pdf]
